# Effect of modified accompanying video education plus continuous nursing on psychological emotion and disease knowledge mastery of patients undergoing endoscopic retrograde cholangiopancreatography

**DOI:** 10.4314/ahs.v25i2.35

**Published:** 2025-06

**Authors:** Xiaohua Xiao, Jing Li

**Affiliations:** 1 Department of Gastroenterology, Affiliated Nanhua Hospital, Hengyang Medical School, University of South China, Hengyang 421002, Hunan Province, China; 2 Endoscopic Center, Affiliated Nanhua Hospital, Hengyang Medical School, University of South China, Hengyang 421002, Hunan Province, China

**Keywords:** endoscopic retrograde cholangiopancreatography, modified accompanying video education, continuous nursing, psychological emotion, disease knowledge mastery

## Abstract

**Objective:**

To evaluate the effect of modified accompanying video education plus continuous nursing on the psychological emotion and disease knowledge mastery of patients undergoing endoscopic retrograde cholangiopancreatography (ERCP).

**Methods:**

A total of 156 patients with choledocholithiasis who underwent ERCP were selected and randomly divided into a control group (n=78) and a modified education group (n=78). General education plus continuous nursing was performed for the control group, while modified accompanying video education plus continuous nursing was conducted for the modified education group. The surgical indicators were observed and the psychological emotion, disease knowledge mastery, self-care ability, health behaviors, self-perceived burden (SPB) and self-efficacy were compared.

**Results:**

After intervention, the scores of Self-rating Depression Scale, Self-rating Anxiety Scale and SPB in the modified education group were lower than those of the control group, while the scores of Exercise of Self-Care Agency Scale, Health Promoting Lifestyle Profile-II, disease knowledge mastery and self-efficacy were higher than those of the control group (P<0.05). After intervention, the modified education group had lower white blood cell count and higher hemoglobin level than those of the control group (P<0.05).

**Conclusion:**

Modified accompanying video education plus continuous nursing can improve the psychological emotion, make the family members more satisfactory.

## Introduction

Stones in the common bile duct are collectively referred to as choledocholithiasis which is a common abdominal disease frequently occurring in the lower segment^1^,^2^. The onset and progression of choledocholithiasis may affect bile excretion and induce liver and bile duct lesions, seriously threatening the health and living quality of patients^3^,^4^. At present, endoscopic retrograde cholangiopancreatography (ERCP) is commonly used for the clinical treatment of choledocholithiasis. Compared with other therapies, ERCP is typified by small trauma and high efficacy, which is conducive to the recovery of patients^5^,^6^. However, most patients with choledocholithiasis and their family members lack ERCP-related knowledge, and the requirements for operating accuracy are high, so high-quality nursing is required^7^,^8^. Correct education for patients undergoing ERCP is helpful to improve both the perioperative compliance and recovery of patients. Continuous nursing is a nursing mode with the advantages of continuity, coordination, comprehensiveness and cooperation.

## Materials and Methods

### Materials

The sample size was determined according to n=µα2×P(1-P)/δ2. The flow diagram for subject enrollment is shown in Figure 1. A total of 156 choledocholithiasis patients who underwent ERCP in our hospital from September 2021 to September 2022 were selected and randomly divided into a control group (n=78) and a modified education group (n=78).

The inclusion criteria were as follows: 1) patients diagnosed with choledocholithiasis by imaging examination in our hospital, 2) those with normal cardiopulmonary functions, no contraindications to ERCP, and no history of ERCP, and 3) those with complete medical data.

The exclusion criteria involved: 1) patients with incomplete medical data, 2) those with communication or consciousness disorders, 3) those with heart, kidney or lung dysfunction, 4) those with mental diseases, 5) those with diseases affecting the pain perception, or 6) those with poor compliance.

### Intervention methods

In the control group, after the patients were admitted to hospital, the nurses assisted the clinicians in the preoperative examination on the patients and explained the preoperative precautions to the patients. General education covering definition of disease, main contents of diagnosis and treatment by ERCP, possible complications, postoperative diet and medication guidance and post-discharge precautions was given.

In the modified education group, a medical nursing team was built, consisting of one chief physician, two associate chief physicians, two attending physicians, two associate chief nurses and three nurses in the Department of Hepatobiliary Surgery of our hospital. After admission, the patients and their family members acquainted themselves with the environment of the ward and operating room under the nurses' guidance and underwent the preoperative examination. Then the nurses prepared the preoperative drugs and explained the precautions for medication to the patients and their family members. Appropriate videos and music were jointly selected by the medical team members and ERCP patients, and combined with videos on ERCP, so that modified accompanying education videos were completed, followed by education for the patients at 3:00-5:00 p.m. on the day before surgery. After the education, the patients were asked to raise questions for 15-20 min, and these questions were answered patiently and in detail. Moreover, a WeChat group was established to facilitate patient-nurse and patient-patient communication. After discharge, continuous nursing was provided in both groups. To be specific, a follow-up team composed of a head nurse and clinical nurses was established, made regular home visits to the ERCP patients, and communicated with the patients and their family members to fully understand the postoperative recovery, daily living conditions and psychological state of the patients. Targeted knowledge education and life and treatment guidance were determined according to different conditions of the patients.

### Surgical indicators

The time of postoperative exhaust, first feeding and ambulation was compared between the two groups.

### Evaluation of psychological emotion

Depression and anxiety were evaluated by the Self-rating Depression Scale (SDS), Self-rating Anxiety Scale (SAS), respectively^9^,^10^. SDS and SAS each include 20 items (1-4 points), and a higher score means more serious depression/anxiety.

### Disease knowledge mastery

A survey was made on the patients using a self-made disease knowledge questionnaire covering disease knowledge, psychological regulation, drug intervention and diet guidance. The highest score of a single item was 100 points, and the score >85 points was considered mastery of disease knowledge.

### Evaluation of self-care ability and health behaviors

The self-care ability of patients was evaluated using the Exercise of Self-Care Agency Scale (ESCA)^11^, with 4 items and a total score of 184 points. The lower the score, the poorer the self-care ability. Health behaviors were assessed by the Health Promoting Lifestyle Profile-II (HPLP-II)^12^, with 52 items in 6 dimensions. A lower total score means poorer health behaviors.

### Evaluation of self-perceived burden (SPB) and self-efficacy

The SPB score^13^ was used to evaluate the patient's SPB, which included 4 items, with the highest score of 16 points, and the lower the score, the lower the burden. The self-efficacy score^14^ was used to evaluate the patient's self-efficacy, which included 10 items (1-4 points), with the highest score of 40 points, and a lower score suggests lower self-efficacy.

### Detection of biochemical indicators

Before and after the intervention, 5 mL of venous blood was drawn from each patient in the morning, and the white blood cell (WBC) count and hemoglobin (Hb) level were detected using an automatic blood cell analyzer.

### Assessment of compliance rate, incidence rate of complications and family satisfaction

The patient's compliance was assessed from such aspects as taking the medicine the doctor prescribed, not abusing and changing drugs privately, and eating, resting and reexamining as advised by the doctor. Complete compliance was defined as adherence to all the above aspects, complete incompliance as adherence to none of the above aspects, and compliance as other situations. The compliance rate was calculated: (complete compliance + compliance)/total cases. Besides, the incidence rate of gastrointestinal bleeding, pancreatitis, abdominal distension and other complications was statistically compared. The family satisfaction was investigated as follows: very satisfied: >90 points, satisfied: 60-90 points, not satisfied: <60 points. Total satisfaction = (very satisfied + satisfied)/total cases.

### Statistical analysis

SPSS 26.0 software was used for statistical analysis. The normality of data was analyzed by the Kolmogorov-Smirnov test. The normally distributed measurement data were described by (x̅ ± s) and compared between two groups by the independent-samples t-test. The count data were described by percentage (%) and compared between two groups by the χ2 test. P<0.05 was considered statistically significant.

## Results

### Baseline clinical data

In the control group, there were 41 males and 37 females aged 25-60 years, with an average of (42.5±13.8) years, and the BMI was (22.51±1.58) kg/m2. One stone was found in 39 cases, two stones in 26 cases, and more than two stones in 13 cases. In the modified education group, there were 43 males and 35 females aged 28-59 years, with an average of (43.2±12.4) years, and the BMI was (22.08±1.33) kg/m2. One stone was found in 41 cases, two stones in 25 cases, and more than two stones in 12 cases. The above general data were balanced and comparable between the two groups (P>0.05) (Table 1).

**Table 1:** Baseline clinical data

Variable		Control group (n=78)	Modified education group (n=78)	*t/x^2^*	*P*
Gender	Male	41	43	0.103	0.748
	Female	37	35		
Age (year)		42.5±13.8	43.2±12.4	0.333	0.739
BMI (kg/m^2^)		22.51±1.58	22.08±1.33	1.839	0.068
History of hypertension		31	28	0.245	0.620
History of diabetes mellitus		26	23	0.268	0.605
Number of stones	1	39	41	0.103	0.749
	2	26	25	0.029	0.864
	>2	13	12	0.048	0.827

### Surgical indicators

The time of postoperative exhaust, first feeding and ambulation of the modified education group was significantly shorter than that of the control group (P<0.05) (Table 2).

**Table 2:** Surgical indicators [(x̅ ± s), h]

Group	Case (n)	Exhaust	First feeding	Ambulation
Control	78	21.53±3.11	17.13±2.65	22.54±3.07
Modified education	78	16.62±2.51	9.58±2.13	14.27±2.35
*t*		10.850	19.610	18.890
P		<0.001	<0.001	<0.001

### Changes in psychological emotion scores

There were 2 no statistically significant differences in the SDS and SAS scores between two groups before intervention (P>0.05). After intervention, the SDS and SAS scores of the modified education group were lower than those of the control group (P<0.05) (Table 3).

**Table 3:** Changes in psychological emotion scores [(x̅ ± s), point]

Group	Case (n)	SDS score		SAS score	

		Before intervention	After intervention	Before intervention	After intervention
Control	78	47.13±5.12	34.39±4.22	48.66±5.36	35.27±4.06
Modified education	78	47.95±5.03	30.15±3.51	49.34±5.12	31.33±3.52
*t*		1.009	6.822	0.810	6.476
P		0.315	<0.001	0.419	<0.001

### Disease knowledge mastery

No statistically significant difference was found in the disease knowledge mastery between the two groups before intervention (P>0.05). After intervention, the scores of disease knowledge mastery of the modified education group were higher than those of the control group (P<0.05) (Table 4).

**Table 4:** Disease knowledge mastery [(x̅ ± s), point]

Group	Case	Disease knowledge		Psychological regulation		Drug intervention		Diet guidance	
	
	(n)	Before intervention	After intervention	Before intervention	After intervention	Before intervention	After intervention	Before intervention	After intervention
Control	78	56.32±6.32	83.17±9.35	62.45±6.37	86.59±9.38	51.33±5.62	85.19±8.50	59.33±6.16	87.11±8.52
Modified education	78	55.64±6.15	92.31±11.52	61.71±6.08	93.62±10.63	50.59±5.31	92.66±9.73	58.52±5.99	95.16±9.78
*t*		0.681	5.441	0.742	4.380	0.845	5.106	0.833	5.481
P		0.497	<0.001	0.459	<0.001	0.399	<0.001	0.406	<0.001

### Changes in self-care ability and health behaviors

Before intervention, the ESCA and HPLP-II scores had no statistically significant differences between the two groups (P>0.05). After intervention, the ESCA and HPLP-II scores of the modified education group were higher than those of the control group (P<0.05) (Table 5).

**Table 5:** Changes in self-care ability and health behaviors [(x̅ ± s), point]

Group	Case	ESCA score		HPLP-II score	
	
	(n)	Before intervention	After intervention	Before intervention	After intervention
Control	78	86.59±9.10	112.37±11.52	17.69±2.61	23.69±2.85
Modified education	78	85.77±8.85	128.97±13.21	17.12±2.35	28.11±3.05
*t*		0.571	8.364	1.433	9.352
P		0.569	<0.001	0.154	<0.001

### Changes in SPB and self-efficacy

Before intervention, no statistically significant differences were found in the SPB and self-efficacy scores between the two groups (P>0.05). After intervention, the modified education group had a lower SPB score and a higher self-efficacy score than those of the control group (P<0.05) (Table 6).

**Table 6:** Changes in SPB and self-efficacy [(x̅ ± s), point]

Group	Case	SPB score		Self-efficacy score	
	
	(n)	Before intervention	After intervention	Before intervention	After intervention
Control	78	14.67±2.33	9.03±1.75	22.96±2.28	28.05±2.66
Modified education	78	15.03±2.08	7.11±1.62	22.33±2.07	31.22±3.37
*t*		1.018	7.111	1.807	6.521
P		0.310	<0.001	0.073	<0.001

### Changes in the levels of biochemical indicators

Before intervention, the WBC count and Hb level had no statistically significant differences between the two groups (P>0.05). After intervention, the WBC count rose and the Hb level declined in both groups, and the modified education group had lower WBC count and higher level of Hb than those of the control group (P<0.05) (Table 7).

**Table 7:** Changes in the levels of biochemical indicators (x̅ ± s)

Group	Case	WBC count (×10^9^/L)		Hb (mg/dL)	
	
	(n)	Before intervention	After intervention	Before intervention	After intervention
Control	78	7.65±1.27	10.78±1.98	138.29±12.12	112.15±10.28
Modified education	78	7.91±1.35	9.15±1.66	136.71±13.51	124.39±11.33
*t*		1.239	5.572	0.769	7.066
P		0.217	<0.001	0.443	<0.001

### Compliance rate, incidence rate of complications and family satisfaction

There was no statistically significant difference in the incidence rate of complications between the two groups (P>0.05). The compliance rate and family satisfaction of the modified education group were superior to those of the control group (P<0.05) (Table 8).

**Table 8:** Compliance rate, incidence rate of complications and family satisfaction [n (%)]

Group	Case (n)	Compliance rate	Incidence rate of complications	Family satisfaction
Control	78	66 (84.62)	8 (10.26)	68 (87.18)
Modified education	78	75 (96.15)	6 (7.69)	76 (97.44)
χ^2^		5.974	0.314	5.778
P		0.015	0.575	0.016

## Discussion

Surgery is the most commonly used means for treating choledocholithiasis in clinic, but the clinical efficacy of routine lithotomy on nearly one fifth of patients with choledocholithiasis is unsatisfactory. In recent years, ERCP has gradually developed to become the most commonly used treatment for choledocholithiasis^15^,^16^. However, most patients undergoing ERCP know little about relevant knowledge, so they are prone to negative emotions during the perioperative period. Given this, effective preoperative education for patients is of great significance for reducing their psychological pressure and improving their compliance^17^,^18^. Modified accompanying videos are jointly selected and produced by the medical staff and ERCP patients and used for pre-treatment education for patients. Continuous nursing is a nursing mode superior in continuity, coordination, comprehensiveness and cooperation, which, combined with modified accompanying video education, can contribute to the treatment and rehabilitation of patients with choledocholithiasis. In this study, the time of postoperative exhaust, first feeding and ambulation of the modified education group was shorter than that of the control group, suggesting that modified accompanying video education can benefit the postoperative recovery of patients.

Most patients with choledocholithiasis suffer anxiety and even fear since they do not well understand ERCP, ERCP will cause psychological stress to patients, and some patients are even fearful about prognosis, thus adversely affecting clinical treatment and recovery^19^,^20^. In this study, choledocholithiasis patients who received modified accompanying video education had significantly decreased SDS and SAS scores and increased scores of disease knowledge mastery, indicating that modified accompanying video education plus continuous nursing can enhance the patient's disease knowledge mastery and effectively relieve anxiety, depression and other negative emotions. Possibly, modified accompanying video education can help patients deeply understand the ERCP-related knowledge, relieve their psychological pressure and enable them to calmly face the disease and ERCP.

Patients with choledocholithiasis need to pour attention into lifestyle and self-care after discharge. Therefore, patients should be guided to develop good health behaviors during rehabilitation and foster self-care ability, forming a virtuous cycle in life after treatment, so that they can take the initiative in the recovery process. In this study, the ESCA and HPLP-II scores rose among patients who received modified accompanying video education plus continuous nursing, suggesting that modified accompanying video education plus continuous nursing can improve the self-care ability of patients, help them develop health behaviors and make them have a strong sense of responsibility. Probably, the combination of the two can help patients understand the precautions after discharge and guide them to carry out self-care, thereby enhancing their self-care ability.

Due to pain resulting from the occurrence and development of choledocholithiasis and psychological stress brought by ERCP, SPB may emerge in patients. Improving patients' self-efficacy can make them take matters into their own hands in the treatment and promote the recovery from disease. In this study, the modified education group had a decreased SPB score and an increased self-efficacy score, suggesting that modified accompanying video education plus continuous nursing can reduce the SPB and enhance the self-efficacy of patients. Taken together, with the use of modified accompanying video education, patients can more fully understand the relevant knowledge of choledocholithiasis and ERCP, reduce SPB while relieving the negative emotions, and establish belief in rehabilitation, thereby enhancing their self-efficacy.

## Conclusion

Modified accompanying video education plus continuous nursing can improve the psychological emotion, health behaviors and self-efficacy, enhance the disease knowledge mastery and self-care ability, reduce the SPB and stress response of patients undergoing ERCP, raise the compliance rate, and make patients' family members more satisfactory.
